# Persistent elevation of postoperative neutrophil-to-lymphocyte ratio: A better predictor of survival in gastric cancer than elevated preoperative neutrophil-to-lymphocyte ratio

**DOI:** 10.1038/s41598-017-13969-x

**Published:** 2017-10-25

**Authors:** Kyueng-Whan Min, Mi Jung Kwon, Dong-Hoon Kim, Byoung Kwan Son, Eun-Kyung Kim, Young Ha Oh, Young Chan Wi

**Affiliations:** 1 Department of Pathology, Hanyang University Guri Hospital, Hanyang University College of Medicine, Guri, Gyeonggi-do Republic of Korea; 2 Department of Pathology, Hallym University Sacred Heart Hospital, Hallym University College of Medicine, Anyang, Gyeonggi-do Republic of Korea; 3 Department of Pathology, Kangbuk Samsung Hospital, Sungkyunkwan University School of Medicine, Seoul, Republic of Korea; 4 Departments of Internal medicine, Eulji Hospital, Eulji University School of medicine, Seoul, Republic of Korea; 5 Department of Pathology, Eulji Hospital, Eulji University School of medicine, Seoul, Republic of Korea

## Abstract

Postoperative neutrophil-to-lymphocyte ratio change (NLRc) reflects the dynamic change of balance between host inflammatory response and immune response after treatment. In gastric cancer, an elevated initial NLR (iNLR) is reported to be a prognostic predictor, but the clinical application of the NLRc remains unclear. The NLRc was assessed in 734 patients undergoing total/subtotal gastrectomy and endoscopic submucosal dissection for gastric adenocarcinoma. The iNLR and NLRc were recorded within 10 days of the first diagnosis and 3–6 months after surgery, respectively. Using receiver operating characteristic (ROC) curves, we investigated the relationship between NLRc or iNLR and patient survival. The analysis revealed a higher predictive power for correlating patient survival with the NLRc compared with iNLR. NLRc was defined as negative (lower than iNLR) and positive (higher than iNLR). A positive NLRc was frequently observed in patients with advanced AJCC stage, local recurrence, distant metastasis, perineural invasion, and adjuvant chemotherapy (all *p* < 0.05). Univariate and multivariate analyses revealed a significant relationship between patient survival and NLRc (all *p* < 0.05) but no association between survival and iNLR. The NLRc could be a better indicator than iNLR for predicting survival in patients with gastric cancer.

## Introduction

Gastric cancer is a complex, heterogeneous disease with a global variation in its aetiology, incidence, natural course, and management. The tumourigenic conditions of gastric cancers are associated with infectious agents (*Helicobacter pylori* or Epstein-Barr virus)^1,2^, lecular subset (*CDH1* mutation)^3^, and different levels of susceptibility and aggressiveness of the tumours. The clinical factors for determining a treatment plan are location of the primary tumour (proximal and distal), subtypes of adenocarcinoma (diffuse, intestinal, or mixed)^4^, tumour susceptibility based on ethnicity, and predictive biomarkers (*HER2*)^5^. Generally, the following prognostic indicators are considered in clinical management: American joint Committee on Cancer (AJCC) stage and size, histological type and grade, and lymphovascular and perineural invasion. A study by the Cancer Genome Atlas Research Network indicated that gastric cancers can be classified into four subtypes based on molecular characteristics: Epstein-Barr virus positivity, microsatellite instability, genomic stability, and chromosomal instability^6^. However, these molecular analyses might be limited in general hospitals that lack facilities. Moreover, molecular markers are expensive and often time-consuming to measure. When there are sufficient technologists with an appropriate laboratory setting, genetic analyses to determine the new subtypes can be easily used in clinical application.

Systemic inflammation is associated with the progression of various cancers by induction of angiogenesis, metastasis and malignant cell proliferation, and alteration of the response to systemic therapy^7^. Neutrophils are known to be key mediators of tumour inflammation^8^ and angiogenesis^9^. Lymphocytes have been suggested to play vital roles in cytotoxic tumour cell death linked to tumour-infiltrating lymphocytes, especially T lymphocytes^10^. The interaction between the cancer and immune system might also extend beyond the local tissue environment. Other systemic inflammatory markers such as C-reactive protein and interleukin-6, have been associated with worse survival in patients with gastric cancer^11^. The imbalance between neutrophils and lymphocytes is thought to be secondary to tumour hypoxia or necrosis and associated with anti-apoptotic effects. Published data demonstrated that the initial neutrophil-to-lymphocyte ratio (iNLR) at that time of first diagnosis is linked to prognosis of different types of cancer^12–15^ as well as of other conditions such as cardiovascular disease^16^ and bacterial infection^17^. Accordingly, the iNLR might represent the balance between pro-tumour inflammatory status and anti-tumour immune status.

Several studies focused primarily on iNLR, while the dynamic change of neutrophil-to-lymphocyte ratio after treatment was not considered. The postoperative neutrophil-to-lymphocyte ratio change (NLRc) might be a meaningful factor to assess prognosis after treatment, because there has been a change in the treatment approach including tumour removal and chemotherapy. However, although the NLRc might dynamically reflect the alteration of balance between host inflammatory response and immune response against cancer after treatment, its significance is largely unclear.

The aim of this study was to investigate the relationships between iNLR and NLRc and clinicopathological parameters, and to evaluate the prognostic value of the NLRc in patients with gastric cancer.

## Results

### Patient characteristics

The median age of the 734 patients was 66 (range: 23–87) years. The following clinicopathological variables were recorded: Sex; age; 8^th^ AJCC stage; location; local recurrence; distant metastasis; size; Lauren type; histological grade; lymphatic, vascular, and perineural invasion; presence of adjuvant chemotherapy; margin involvement, and patient survival.

EGC was present in 395 patients and advanced gastric cancer (AGC) in 339. In early gastric cancer (EGC), the predominant gross type was classified as protruding (type I) in 21 patients, slightly elevated (type IIa) in 28, flat (type IIb) in 113, slightly depressed (type IIb) in 196, and excavated (type III) in 37. In AGC, the predominant gross type was classified as fungating (Borrmann type 1) in 11 patients, ulcerative (Borrmann type 2) in 66, ulcero-infiltrative (Borrmann type 3) in 221, and linitis plastica (Borrmann type 4) in 41. According to the Lauren classification, 396 patients had intestinal type, followed by 217 with a diffuse type and 121 with a mixed type. The surgical procedures included subtotal gastrectomy in 567, total gastrectomy in 106, endoscopic submucosal dissection in 54, and endoscopic mucosal resection in seven. Two hundred thirty-one patients received adjuvant chemotherapy such as tegafur-uracil. After surgery, 142 patients developed local recurrence and/or new distant metastases, and 172 patients died during the median follow-up period of 49.41 months.

### Evaluation of the neutrophil-to-lymphocyte ratio

**Figure 1:**
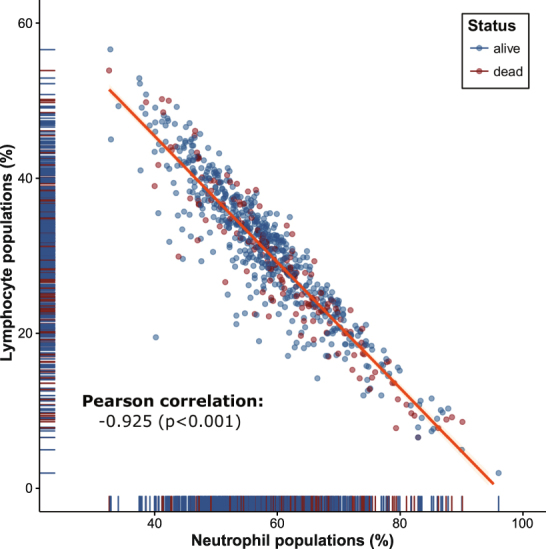
Scatter plot of lymphocytes and neutrophils. The neutrophils population (%) is inversely proportional to the lymphocyte populations. There is a correlation between neutrophil and lymphocyte populations in the validation set using Pearson’s correlation (−0.925, *p* < 0.001) (alive: blue, dead: red).

In comparison of systemic inflammation response markers, there was a negative association between absolute neutrophil counts and absolute lymphocyte counts (Fig. 1).

**Figure 2:**
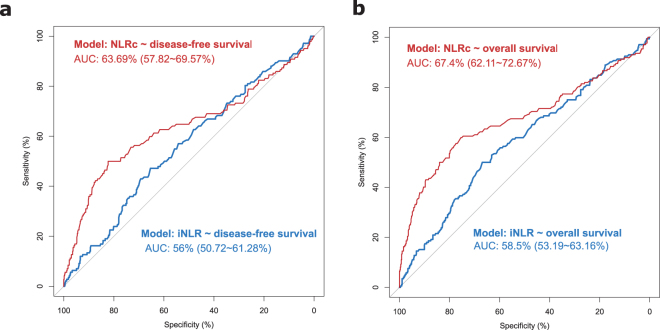
Receiver operating characteristic (ROC) curve showing the statistical performance of the initial neutrophil-to-lymphocyte ratio (iNLR) (**a**) and the postoperative neutrophil-to-lymphocyte ratio change (NLRc) (**b**) depending on the disease-free and overall survival rate.

We subsequently analysed survival using receiver operating characteristic (ROC) curves to evaluate the probability of iNLR and NLRc according to patient survival. The ROC curve revealed that the NLRc (area under the ROC curve, 0.674; sensitivity, 59.9%; specificity, 75.4%; positive predictive value, 14%, negative predictive value, 57.3%) had a greater predictive power for determining OS than the iNLR (area under the ROC curve, 0.584; sensitivity, 50%; specificity, 66.9%; positive predictive value, 18.6%, negative predictive value, 68.4%). Using DFS as the end point, the area under the ROC curve for the NLRc (area under the ROC curve, 0.637; sensitivity, 50%; specificity, 82.3%; positive predictive value, 12.7%, negative predictive value, 59.7%) was higher than that for iNLR (area under the ROC curve, 0.560; sensitivity, 47.2%; specificity, 65.4%; positive predictive value, 16.2%, negative predictive value, 75.4%) (Fig. 2).

The NLRc was defined as negative (NLRc ≤ 0) when the postoperative NLR was lower than the preoperative NLR. The NLRc was defined as positive (NLRc > 0) when the postoperative NLR was higher than the preoperative NLR.

### Associations between the neutrophil-to-lymphocyte ratio change and clinicopathological parameters

We analysed clinicopathological differences between patients with a negative and positive NLRc. The positive NLRc correlated to advanced AJCC stage (I, II *vs*. III; OR, 1.57; 95% CI: 1.12–2.2; *P* = 0.008), high T stage (1 *vs*. 2, 3, 4; OR, 1.39; 95% CI, 1.02–1.88; *P* = 0.037), high N stage (0, 1 *vs*. 2, 3; OR, 1.69; 95% CI, 1.21–2.36; *P* = 0.002), local recurrence (OR: 3.44, 95% CI: 1.55–7.62, *P* = 0.001), distant metastasis (OR: 2.79, 95% CI: 1.83–4.05, *P* < 0.001), perineural invasion (OR: 1.57, 95% CI: 1.08–2.29, *P* = 0.019), and administration of adjuvant chemotherapy (OR: 1.55, 95% CI: 1.125–2.15, *P* = 0.007) (Table 1). In addition, a positive NLRc was frequently observed in patients with AGC than in those with EGC (OR, 1.41; 95% CI, 1.04–1.91; *P* = 0.028). In comparisons of clinicopathological parameters between AGC and EGC, AGC was associated with high N stage (0, 1 *vs*. 2, 3; OR, 46.7; 95% CI, 24.7–88.29; *P* < 0.001), large tumour size (≤3 *vs*. >3 cm; OR, 10.9; 95% CI, 7.62–15.59; *P* < 0.001), tumour location (cardia fundus, body *vs*. antrum, pylorus; OR, 0.68; 95% CI, 0.51–0.91; *P* = 0.009), advanced histological grade (well, moderately *vs*. poorly, signet ring; OR, 2.52; 95% CI, 1.87–3.39; *P* < 0.001), diffuse type (OR, 2.26; 95% CI, 1.63–3.13; *P* < 0.001), lymphovascular (OR, 9.41; 95% CI, 6.68–13.27; *P* < 0.001) and perineural invasion (OR, 34.9; 95% CI, 16.02–76.06; *P* < 0.001), and administration of adjuvant chemotherapy (OR, 2.55; 95% CI, 1.85–3.5; *P* < 0.001).

**Table 1:** Association between clinicopathological parameters and the postoperative neutrophil-to-lymphocyte ratio change (NLRc) in 734 patients with gastric cancer.

Parameters	N = 734	NLRc		*p*-value (Chi-square)
		Negative (n = 483), %	Positive (n = 251), %	
Sex				
Male	499	322 (66.7)	177 (70.5)	0.289
Female	235	161 (33.3)	74 (29.5)	
Age (year)				
≤ 65	366	250 (51.8)	116 (46.2)	0.154
>65	368	233 (48.2)	135 (53.8)	
AJCC stage				
I	418	285 (59)	133 (53)	**0 008** ^1^
II	115	81 (16.8)	34 (13.5)	
III	201	117 (24.2)	84 (33.5)	
T criteria				
1	393	272 (56.3)	121 (48.2)	**0 037** ^2^
2	64	40 (8.3)	24 (9.6)	
3	171	104 (21.5)	67 (26.7)	
4	106	67 (13.9)	39 (15.5)	
N criteria				
0	445	307 (63.6)	138 (55)	**0 002** ^3^
1	84	59 (12.2)	25 (10)	
2	72	51 (10.6)	21 (8.4)	
3	133	66 (13.70	67 (26.7)	
Tumour location				
Cardia or fundus	37	25 (5.2)	12 (4.8)	0.272^4^
Body	317	215 (44.5)	102 (40.6)	
Antrum or pylorus	380	243 (50.3)	137 (54.6)	
Local recurrence				
Absence	707	473 (97.9)	234 (93.2)	**0 001**
Presence	27	10 (2.1)	17 (6.8)	
Distant metastasis				
Absence	614	428 (88.6)	186 (74.1)	**<0 001**
Presence	120	55 (11.4)	65 (25.9)	
Tumour size				
≤3 cm	323	221 (45.8)	102 (40.6)	0.185
>3 cm	411	262 (54.2)	149 (59.4)	
Lauren type				
Intestinal	396	271 (56.1)	125 (49.8)	0.066^5^
Mixed	217	132 (27.3)	85 (33.9)	
Diffuse	121	80 (16.6)	41 (16.3)	
Histological grade				
Well	135	98 (20.3)	37 (14.7)	0.06^6^
Moderately	225	151 (31.3)	74 (29.5)	
Poorly	266	161 (33.3)	105 (41.8)	
Signet ring	108	73 (15.1)	35 (13.9)	
Lymphatic invasion				
Negative	437	291 (60.2)	146 (58.2)	0.586
Positive	297	192 (39.8)	105 (41.8)	
Vascular invasion				
Negative	640	423 (87.6)	217 (86.5)	0.666
Positive	94	60 (12.4)	34 (13.5)	
Perineural invasion				
Negative	596	404 (83.6)	192 (76.5)	**0 019**
Positive	138	79 (16.4)	59 (23.5)	
Adjuvant chemotherapy				
absence	503	347 (71.8)	156 (62.2)	**0 007**
presence	231	136 (28.2)	95 (37.8)	
Margin				
Uninvolved	714	472 (97.7)	242 (96.4)	0.302
involved	20	11 (2.3)	9 (3.6)	

AJCC, American Joint Committee on Cancer, 8th edition.

^1^I, II versus III.

^2^T 1 versus 2, 3, 4.

^3^N 0, 1 versus 2, 3.

^4^Cardia, fundus, body versus antrum, pylorus.

^5^ intestinal, mixed versus diffuse.

^6^well, moderately versus poorly, signet ring.

*p* < 0.05 is shown in bold.

### Differences in disease-free and overall survival according to the neutrophil-to-lymphocyte ratio change

Based on previous studies^18,19^, the cut-off values of iNLR were <3 and ≥3. The iNLR was significantly related to overall survival (OS) (HR, 1.74; 95% CI, 1.22–2.48; *P* = 0.002), but not to disease-free survival (DFS) (HR, 1.38; 95% CI, 0.91–2.09; *p* = 0.128). After adjustment for confounders including age, T and N criteria, lymphovascular and perineural invasion, and margin involvement, there was no significant relationship between iNLR and DFS or OS (multivariate DFS HR, 0.8; 95% CI, 0.52–1.23; *p* = 0.306 and OS HR, 1.07; 95% CI, 0.74–1.54; *p* = 0.732) (Table 2).

**Table 2:** Disease-free and overall survival analyses according to initial neutrophil-to-lymphocyte ratio (iNLR) and neutrophil-to-lymphocyte ratio change (NLRc) in 734 patients with gastric cancer.

Survival	Univariate^1^	Multivariate^2^	HR	95% CI	
				Lower	Upper
**Disease-free survival**					
iNLR (<3 vs. ≥3)	0.128	0.306	0.8	0.523	1.226
Age (≤65 vs. >65)	0.129	0.44	1.143	0.815	1.602
T criteria (1, 2 vs. 3, 4)	**<0.001**	**<0.001**	5.31	3.036	9.289
N criteria (0, 1 vs. 2, 3)	**<0.001**	**<0.001**	2.334	1.489	3.659
Lymphovascular invasion (absence vs. presence)	**<0.001**	**0.019**	1.663	1.088	2.542
Perineural invasion (absence vs. presence)	**<0.001**	0.147	1.325	0.906	1.938
Margin involvement (absence vs. presence)	0.124	0.086	2.216	0.894	5.492
**Disease-free survival**					
NLRc (negative vs. positive)	**<0.001**	**<0.001**	2.533	1.802	3.562
Age (≤65 vs. >65)	0.129	0.837	1.036	0.74	1.45
T criteria (1, 2 vs. 3, 4)	**<0.001**	**<0.001**	5.412	3.092	9.472
N criteria (0, 1 vs. 2, 3)	**<0.001**	**0.003**	2	1.269	3.153
Lymphovascular invasion (absence vs. presence)	**<0.001**	**0.002**	1.946	1.269	2.986
Perineural invasion (absence vs. presence)	**<0.001**	0.502	1.141	0.776	1.678
Margin involvement (absence vs. presence)	0.124	0.178	1.87	0.752	4.65
**Overall survival**					
iNLR (<3 vs. ≥3)	**0.002**	0.732	1.066	0.739	1.537
Age (≤65 vs. >65)	**0.001**	**0.005**	1.559	1.142	2.127
T criteria (1, 2 vs. 3, 4)	**<0.001**	**0**	3.466	2.202	5.457
N criteria (0, 1 vs. 2, 3)	**<0.001**	**0.001**	1.995	1.331	2.991
Lymphovascular invasion (absence vs. presence)	**<0.001**	0.053	1.44	0.995	2.085
Perineural invasion (absence vs. presence)	**<0.001**	0.38	1.179	0.816	1.702
Margin involvement (absence vs. presence)	**0.001**	**0.001**	3.131	1.582	6.196
**Overall survival**					
NLRc (negative vs. positive)	**<0.001**	**<0.001**	1.448	1.062	1.974
Age (≤65 vs. >65)	**0.001**	0.019	1.448	1.062	1.974
T criteria (1, 2 vs. 3, 4)	**<0.001**	**<0.001**	3.55	2.254	5.591
N criteria (0, 1 vs. 2, 3)	**<0.001**	0.013	1.68	1.114	2.532
Lymphovascular invasion (absence vs. presence)	**<0.001**	**0.003**	1.759	1.214	2.548
Perineural invasion (absence vs. presence)	**<0.001**	0.823	1.043	0.72	1.512
Margin involvement (absence vs. presence)	**0.001**	**0.006**	2.639	1.328	5.245

^1^Log rank test.

^2^Cox proportional hazard model.

*p* < 0.05 is shown in bold.

**Figure 3:**
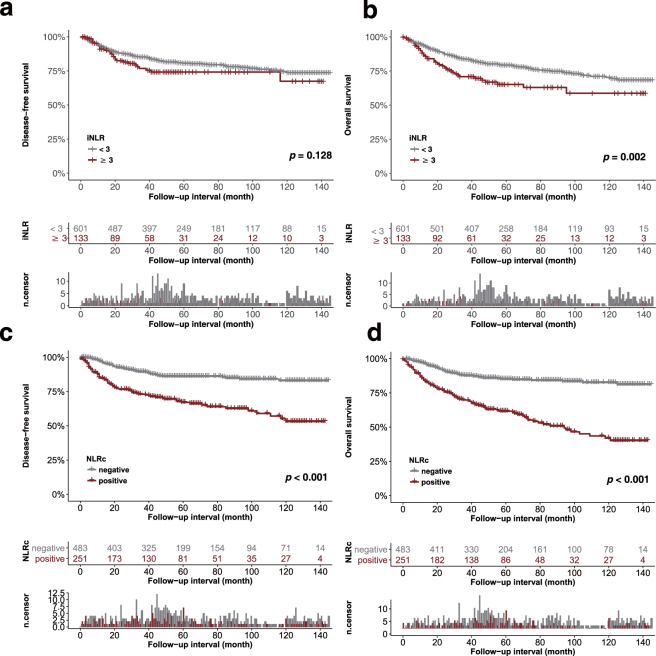
Disease-free and overall survival analysis according the initial neutrophil-to-lymphocyte ratio (iNLR) (**a**,**b**) and postoperative neutrophil-to-lymphocyte ratio change (NLRc) (**c**,**d**). Abbreviations: n.censor, number of censored subjects.

In univariate analyses of the NLRc, there was a significant difference in DFS (HR, 2.97; 95% CI, 2.13–4.14; *p* < 0.001) and OS (HR, 3.55; 95% CI, 2.61–4.81; *p* < 0.001) between the negative and positive groups. In multivariate analyses, positive NLRc was associated with worse DFS (HR, 2.53; 95% CI, 1.8–3.56; *p* < 0.001) and OS (HR, 1.45; 95% CI, 1.06–1.97; *p* < 0.001) (Fig. 3).

**Figure 4:**
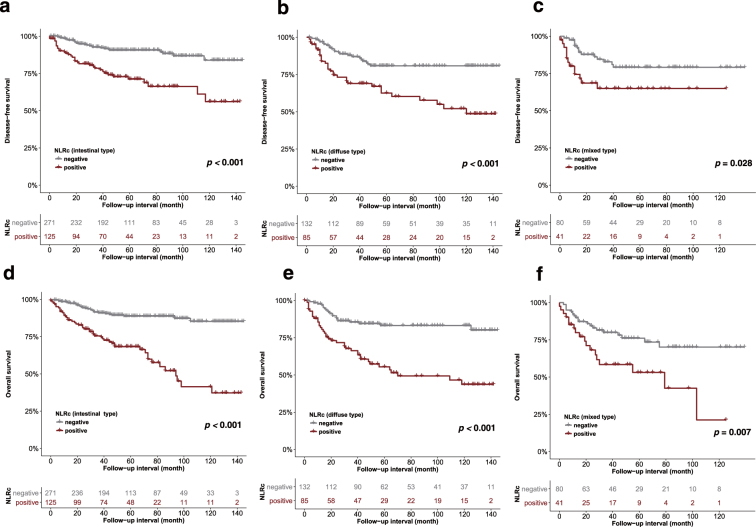
Disease-free survival (**a**,**b**,**c**) and overall survival (**d**,**e**,**f**) curves showing the association between the postoperative neutrophil-to-lymphocyte ratio change (NLRc) according to the Lauren classification, which includes intestinal, diffuse, and mixed type (all *p* < 0.05).

In univariate and multivariate analyses according to the Lauren classification, a positive NLRc was associated with poor DFS and OS in patients with intestinal (univariate DFS HR, 3.4; 95% CI, 2.04–5.65; *p* < 0.001 and OS HR, 4.34; 95% CI, 2.72–6.95; *p* < 0.001; multivariate DFS HR, 3.25; 95% CI, 1.91–5.52; *p* < 0.001 and OS HR, 4.56; 95% CI, 2.81–7.4; *p* < 0.001), diffuse (univariate DFS HR, 2.78; 95% CI, 1.62–4.79; *p* < 0.001 and OS HR, 3.53; 95% CI, 2.1–6.01; *p* < 0.001; multivariate DFS HR, 2.52; 95% CI, 1.42–4.49; *p* < 0.001 and OS HR, 2.92; 95% CI, 1.67–5.1; *p* = 0.002), or mixed type (univariate DFS HR, 2.28; 95% CI, 1.1–4.86; *p* = 0.028 and OS HR, 2.39; 95% CI, 1.25–4.56; *p* = 0.007; multivariate DFS HR, 2.52; 95% CI, 1.1–5.79; *p* = 0.013 and OS HR, 2.45; 95% CI, 1.21–4.97; *p* = 0.029) (Fig. 4).

**Figure 5:**
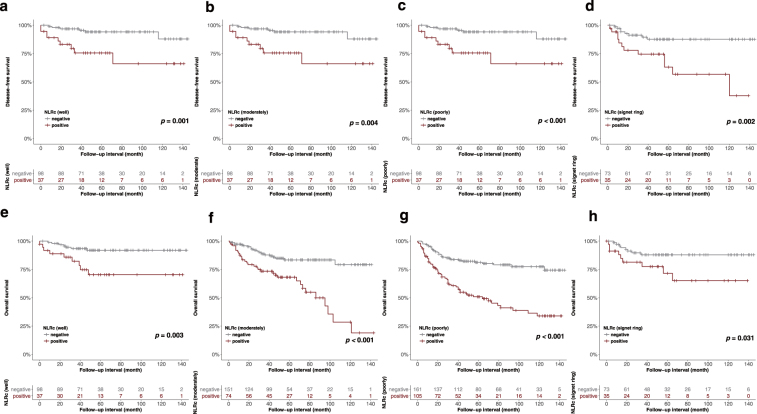
Disease-free survival (**a**,**b**,**c**,**d**) and overall survival (**e**,**f**,**g**,**h**) showing the association between the postoperative neutrophil-to-lymphocyte ratio change (NLRc) according to the histological grade, which includes well, moderately, poorly differentiated and signet ring cell type (all *p* < 0.05).

In univariate and multivariate analyses according to histological grade, there were significant associations between NLRc and DFS and OS in patients with well (univariate DFS HR, 4.84; 95% CI, 1.72–13.64; *p* = 0.001 and OS HR, 4.01; 95% CI, 1.49–10.77; *p* = 0.003; multivariate DFS HR, 11.55; 95% CI, 2.89–46.15; *p* = 0.001 and OS HR, 5.82; 95% CI, 1.76–19.22; *p* = 0.004), poorly differentiated (univariate DFS HR, 2.52; 95% CI, 1.57–4.04; *p* < 0.001 and OS HR, 3.42; 95% CI, 2.2–5.29; *p* < 0.001; multivariate DFS HR, 2.36; 95% CI, 1.45–3.85; *p* = 0.001 and OS HR, 2.91; 95% CI, 1.86–4.56; *p* < 0.001), or signet ring cell types (univariate DFS HR, 3.83; 95% CI, 1.56–9.38; *p* = 0.002 and OS HR, 2.73; 95% CI, 1.05–7.08; *p* = 0.031; multivariate DFS HR, 3.94; 95% CI, 1.53–10.1; *p* = 0.004 and OS HR, 2.74; 95% CI, 1.01–7.42; *p* = 0.048). In univariate analyses of moderate histological grade, the NLRc was statistically related to DFS and OS (DFS HR, 2.48; 95% CI, 1.3–4.74; *p* = 0.004 and OS HR, 3.39; 95% CI, 1.94–5.91; *p* < 0.001). In multivariate analyses of moderate histological grade, the NLRc was statistically related to OS (HR, 3.84; 95% CI, 2.14–6.88; *p* < 0.001) but not to DFS (HR, 1.94; 95% CI, 0.98–3.83; *p* = 0.057) (Fig. 5).

In univariate and multivariate analyses according to adjuvant chemotherapy, there were significant associations between the NLRc and DFS or OS in patients receiving adjuvant chemotherapy (univariate DFS HR, 5.93; 95% CI, 3.3–10.64; *p* < 0.001 and OS HR, 4.62; 95% CI, 2.74–7.79; *p* < 0.001; multivariate DFS HR, 6.36; 95% CI, 3.47–11.67; *p* < 0.001 and OS HR, 4.93; 95% CI, 2.87–8.47; *p* < 0.001). In univariate analyses of patients not receiving adjuvant chemotherapy, the NLRc was linked to poor DFS and OS (DFS HR, 1.77; 95% CI, 1.14–2.76; *p* = 0.011 and OS HR, 2.9; 95% CI, 1.97–4.27; *p* < 0.001). In multivariate analyses, a positive NLRc was associated with poor OS (HR, 4.93; 95% CI, 2.87–8.47; *p* < 0.001) but not with worse DFS, (HR, 1.13; 95% CI, 0.71–1.8; *p* = 0.602) (Table 3).

**Table 3:** Disease-free and overall survival analyses according to the NLR change based on the Lauren classification.

Survival	Univariate^1^	Multivariate^2^	HR	95% CI	
				Lower	Upper
**Disease-free survival**					
Lauren classification					
Intestinal type	**<0.001**	**<0.001**	3.248	1.913	5.516
Diffuse type	**<0.001**	**<0.001**	2.524	1.418	4.493
Mixed type	**0.028**	**0.013**	2.522	1.098	5.79
Histological grade					
Well	**0.001**	**0.001**	11.545	2.888	46.146
Moderately	**0.004**	0.057	1.938	0.98	3.834
Poorly	**<0.001**	**0.001**	2.363	1.453	3.845
Signet ring	**0.002**	**0.004**	3.935	1.534	10.096
Adjuvant chemotherapy					
Not received	**0.01**	0.602	1.131	0.712	1.797
Received	**<0.001**	**<0.001**	6.36	3.466	11.67
**Overall survival**					
Lauren classification					
Intestinal type	**<0.001**	**<0.001**	4.562	2.812	7.4
Diffuse type	**<0.001**	**0.002**	2.923	1.672	5.107
Mixed type	**0.007**	**0.029**	2.453	1.212	4.966
Histological grade					
Well	**0.003**	**0.004**	5.82	1.763	19.22
Moderately	**<0.001**	**<0.001**	3.838	2.141	6.879
Poorly	**<0.001**	**<0.001**	2.91	1.857	4.558
Signet ring	**0.031**	**0.048**	2.736	1.009	7.423
Adjuvant chemotherapy					
Not received	**<0.001**	**<0.001**	2.257	1.517	3.356
Received	**<0.001**	**<0.001**	4.934	2.873	8.473

^1^log rank test.

^2^Cox proportional hazard model; adjusted for age, T and N criteria, lymphovascular and perineural invasion, and margin status.

*p* < 0.05 is shown in bold.

^1^log rank test.

^2^Cox proportional hazard model; adjusted for age, T and N criteria, lymphovascular and perineural invasion, and margin status.

*p* < 0.05 is shown in bold.

## Discussion

The systemic inflammatory reaction in the tumour microenvironment is essential for cancer growth and development. The concept of inflammation-based scores has been applied in many types of cancer. Neutrophils are thought to play a key role in normal physiological angiogenesis and tumour angiogenesis^20,20^. Activated neutrophils can release matrix metalloproteinases (MMPs), in particular MMP-9, which activate potent angiogenic factors (vascular endothelial growth factor, fibroblast growth factor-2)^21,22^. The increase in neutrophil count leads to induction of tumour progression and development of metastases via secretion of cytokines (tumor necrosis factor, IL-1, IL-6)^23,24^. In contrast, lymphocytes are a major factor for the suppression of cancer progression. Cytotoxic lymphocytes, which are ultimately responsible for killing tumour cells and eradicating the tumour, are applicable to cancer immunotherapy^25^. Based on the opposite functions of neutrophils and lymphocytes, the NLR might provide improved prognostic information regarding cancer progression.

The NLRc is a readily available and inexpensive biomarker for differential types of cancer. Nevertheless, the precise mechanisms by which the relationship between neutrophils and lymphocytes might predict clinical outcomes are not fully understood. A study by El-Hag *et al*. demonstrated that neutrophils lead to suppression of the cytolytic activity of immune cells such as lymphocytes, natural killer cells, and activated T cells^26^. In our results, a negative correlation between neutrophils and lymphocytes supports the above concept. Other published data have described several mechanisms in detail as follows: First, the anticancer responses of natural killer cells and activated T cells might be suppressed by marked neutrophil infiltration around the tumour^27^. Therefore, a high NLR might decrease the effects of the lymphocyte-mediated cellular immune response and promote cancer progression. Second, circulating neutrophils contribute to tumour growth and progression by producing cytokines. Considering the complexity and heterogeneity of cancer, the neutrophil count alone might not reflect a decreased lymphocyte-mediated immune response, and a low lymphocyte count alone might not reflect the neutrophil-driven tumour growth process. In addition, a study by Kobayashi *et al*. suggested that the NLR represents the relative extent of inflammation and host immunity against cancer, as its value is directly affected by the total count of neutrophils and lymphocytes. The combined effects of neutrophilia and lymphocytopenia might be better than the effect of a single marker for predicting clinical outcome.

In gastric cancer, a previous study reported the role of the iNLR without evaluation of the NLRc^14,18,19^. These studies focused only on the iNLR, not on the dynamic changes in the NLR after treatment. In different types of cancer, few studies have evaluated dynamic changes in the NLR after treatment. In studies of renal cell carcinoma, a negative NLRc was associated with favourable outcomes^28^. Another study demonstrated that the NLRc is related to clinicopathological parameters such as recurrence, tumour size, and stage^29^. In studies of non-small cell lung cancer and urothelial carcinoma, the NLRc had a greater statistical power than the iNLR in evaluating patient survival^30,31^. A positive NLRc indicates that the microenvironment supporting cancer growth is persistent, despite removing the causal risk factor related to inflammation after cancer treatment. Therefore, the NLRc after treatment is more meaningful than the iNLR at the time of the first diagnosis, because the NLRc reflects the dynamic change of the immune response after treatment. In our study, a positive NLRc had more statistical power than iNLR in predicting DFS and OS.

In summary, the NLRc is statistically associated with increased mortality rates in patients with gastric cancer and has better statistical power than the iNLR. Compared to molecular markers, the NLRc seems to be a convenient, easily obtainable and repeatable, low cost, and reliable predictor for patients with gastric cancer. Practically, this concept of the NLRc could provide clear, concise, and easily applicable information for evaluating prognosis in gastric cancer, which comprises a heterogeneous cancer cell population. Larger scale studies are required to better assess the relationships between the NLRc and prognosis of other types of cancer.

## Materials and Methods

### Patient selection

This retrospective study included 734 patients diagnosed with gastric adenocarcinoma at two hospitals between 2000 and 2013. The Reporting Recommendations for Tumor Marker Prognostic Studies (REMARK) criteria were followed throughout this study. The inclusion criteria were: 1) patients with histopathological evidence of primary adenocarcinoma confirmed by pathologists, and known clinical outcome; and 2) patients with postoperative follow-up blood samples, which were collected in ethylenediaminetetraacetic acid-containing tubes according to other published studies^32^. The exclusion criteria were: patients diagnosed with adenocarcinoma but with inadequate clinical history and/or no available microscopic slides.

### Interpretation of the neutrophil-to-lymphocyte ratio

Total white blood cells (WBCs), neutrophils, and lymphocytes were obtained by complete blood counts before surgery. A follow-up blood sample was obtained at 3–6 months after surgery.

The absolute neutrophil and lymphocyte counts were calculated by multiplying the percentage of each component by number of WBCs. The neutrophil-to-lymphocyte ratio was determined from the differential count by dividing the absolute neutrophil count by the absolute lymphocyte count. To evaluate the NLRc, we subtracted the postoperative NLR from the preoperative NLR: postoperative NLR – preoperative NLR = NLR change.

### Statistical analysis

The associations between clinicopathological parameters and the NLRc were analysed by the chi-square test. The DFS time was defined as the time from the date of diagnosis to the date of local recurrence or new distant metastasis. The OS time was defined as the time from the date of diagnosis to all-cause death. Survival curves were generated using the Kaplan-Meier method and were compared by the log rank test. A Cox regression model was used for multivariate analysis. A p-value < 0.05 was considered statistically significant. All statistical analyses were performed using SPSS statistics (version 20.0, Chicago, IL, USA) and R packages (http://www.r-project.org/).

### Ethics approval

This study (involving human participants) was approved by the Ethics Committee of the Eulji Hospital (EuljiIRB 15–62), and performed with respect to the ethical standards of the Declaration of Helsinki, as revised in 2008. The IRB review confirmed that the informed consent is not necessary in this study.
